# Copper-ATSM as a Treatment for ALS: Support from Mutant SOD1 Models and Beyond

**DOI:** 10.3390/life10110271

**Published:** 2020-11-04

**Authors:** Sara Nikseresht, James B.W. Hilton, Kai Kysenius, Jeffrey R. Liddell, Peter J. Crouch

**Affiliations:** 1 Department of Pharmacology and Therapeutics, The University of Melbourne, Melbourne, VIC 3010, Australia; sara.nikseresht@unimelb.edu.au (S.N.); james.hilton@unimelb.edu.au (J.B.H.); jliddell@unimelb.edu.au (J.R.L.); 2 Department of Pharmacology and Therapeutics and Florey Institute of Neuroscience and Mental Health, The University of Melbourne, Melbourne, VIC 3010, Australia; kai.kysenius@unimelb.edu.au

**Keywords:** amyotrophic lateral sclerosis (ALS), motor neurone disease (MND), copper, copper-ATSM, Cu^II^(atsm), sporadic, neurodegenerative disease

## Abstract

The blood–brain barrier permeant, copper-containing compound, Cu^II^(atsm), has successfully progressed from fundamental research outcomes in the laboratory through to phase 2/3 clinical assessment in patients with the highly aggressive and fatal neurodegenerative condition of amyotrophic lateral sclerosis (ALS). The most compelling outcomes to date to indicate potential for disease-modification have come from pre-clinical studies utilising mouse models that involve transgenic expression of mutated superoxide dismutase 1 (SOD1). Mutant SOD1 mice provide a very robust mammalian model of ALS with high validity, but mutations in SOD1 account for only a small percentage of ALS cases in the clinic, with the preponderant amount of cases being sporadic and of unknown aetiology. As per other putative drugs for ALS developed and tested primarily in mutant SOD1 mice, this raises important questions about the pertinence of Cu^II^(atsm) to broader clinical translation. This review highlights some of the challenges associated with the clinical translation of new treatment options for ALS. It then provides a brief account of pre-clinical outcomes for Cu^II^(atsm) in SOD1 mouse models of ALS, followed by an outline of additional studies which report positive outcomes for Cu^II^(atsm) when assessed in cell and mouse models of neurodegeneration which do not involve mutant SOD1. Clinical evidence for Cu^II^(atsm) selectively targeting affected regions of the CNS in patients is also presented. Overall, this review summarises the existing evidence which indicates why clinical relevance of Cu^II^(atsm) likely extends beyond the context of cases of ALS caused by mutant SOD1.

## 1. Amyotrophic Lateral Sclerosis

Amyotrophic lateral sclerosis (ALS) is a ruinous progressive neurodegenerative disorder which selectively affects motor neurones within the central nervous system (CNS) [1]. Diagnosis relies heavily on the manifestation of clinical symptoms that arise and subsequently worsen as the number of functional motor neurones declines. Patients are primarily diagnosed via symptoms involving a decrease in muscle strength, and most will die due to respiratory failure within 3–5 years. Symptom onset is less likely before the age of 50 or after the age of 70 [2]. The prevalence of ALS ranges between 4.1–8.4 per 100,000 persons, and has been increasing in recent years [2]. Age, male sex, family background, lifestyle, environmental toxins and occupation are all implicated as risk factors, but for the vast majority of cases the aetiology is unknown [3].

There are only two approved therapeutic interventions for ALS and neither has a dramatic disease-modifying effect. Riluzole, the first ALS drug approved by the FDA in 1995 and current first-line treatment worldwide, is a glutamate antagonist that mitigates neuronal degeneration triggered by glutamate excitotoxicity [4]. Edaravone, the second FDA approved treatment for ALS in 2017, is a potent free-radical scavenger and protects cells against oxidative stress. The latter is a newly introduced neuroprotective agent yet to gain widespread regulatory approval [5,6]. While there is no cure for ALS, numerous potential therapeutic drugs including masitinib [7] and methylcobalamin [8], and therapeutic methods including stem cell [9] and gene therapies [10] are currently being investigated in pre-clinical studies and clinical trials.

## 2. Genetic Causes of ALS

Although most ALS cases are sporadic, approximately 10% are familial. The first gene mutation associated with ALS was Cu/Zn-superoxide dismutase (*SOD1*), with 20% of familial ALS cases and 3% of sporadic ALS cases linked to *SOD1* mutations [11,12]. This discovery ultimately led to new approaches to treating ALS in subsequent decades. For example, antisense oligonucleotides have been designed to inhibit SOD1 expression in SOD1 mutation carriers [13,14]. Antisense oligonucleotide therapy is also being studied for patients with mutation in the chromosome nine open reading frame 72 (*C9orf72*) gene [15,16]. Mutation in the *C9orf72* gene (GGGGCC hexanucleotide repeat expansion in the first intron) affects approximately 40% of familial and 10% of sporadic ALS cases [17,18]. Additional, less common genetic causes of ALS include mutations in *TARDPB*, *FUS, ATXN2, ANG*, *SQSTM1/p62*, *DCTN1*, *VAPB*, *VCP*, *DAO*, *OPTN*, *UBQLN2* and *PFN1* [19,20,21,22]. Identification of ALS susceptibility genes is growing and some additional genes have been recently introduced such as *MATR3*, *CHCHD10*, *TBK1*, *TUBA4A*, *NEK1*, *C21orf2*, *CCNF* and *KIF5A* [23,24].

## 3. A Clinical Translation Challenge

The discovery of genetic causes of ALS enabled the generation of animal models based on explicit disease-causing mutations which, in turn, had a dramatic impact on studying disease mechanisms and on pre-clinical assessment of potential therapeutic compounds. Likely reflecting timelines associated with the discovery of *SOD1* mutations in ALS, mutant SOD1 mouse models of the disease provide the most well established and broadly used models, with transgenic mice expressing high levels of human mutant SOD1 under control of the natural *SOD1* gene promoter [25] developing a robust ALS-like phenotype. Substitution of alanine for glycine at position 93 of *SOD1* (SOD1G93A), for example, is a mutation which causes ALS in people [12] and transgenic expression of SOD1G93A in mice induces paralysis and premature mortality [25]. These animals demonstrate motor neurone degeneration whilst SOD1 activity is retained [26]. Glutamate mishandling [27], endoplasmic reticulum stress [28], mitochondrial dysfunction [29], bioenergetic defects [30], axonal transport perturbation [31], and oxidative stress [32] are early consequences of mutant SOD1 expression in SOD1G93A transgenic mice which eventually result in progressive muscle atrophy [33] and motor neurone loss in the cortex, brainstem and spinal cord [34]. Other transgenic rodents expressing different forms of mutant SOD1 have also facilitated research in this area (e.g., G37R, H46R, G85R, G86R, D90A, L126Z, and G127X) [35,36].

By utilising mutant SOD1 mouse models of ALS, the past 20 years have included assessment of a large and diverse number of treatments targeting excitotoxicity, oxidative stress, inflammation, mitochondrial dysfunction, abnormal protein aggregation and dysregulated metabolism [37]. Many of these treatment strategies for ALS have provided promising published outcomes, but successful translation to clinically impactful outcomes has proven to be elusive [38]. This has led to questions regarding validity of the mutant SOD1 models when assessing treatments for a disease which has no known genetic basis for most afflicted individuals. However, because many of these therapeutic targets are evident in sporadic ALS (and even other neurodegenerative diseases), questions must also be asked of the treatments themselves or, more broadly, the potential to extrapolate outcomes from pre-clinical studies that relate to such a fast-progressing neurodegenerative condition.

For example, excitotoxicity is a feature of all forms of ALS and other neurodegenerative diseases [39]. This places excitotoxicity as a therapeutic target with broad appeal that extends well beyond the boundaries of mutant SOD1-related neuronal death. Targeting excitotoxicity using ceftriaxone or memantine produced positive outcomes in mutant SOD1 mice which included delayed loss of muscle strength and weight loss, and lengthened life span, even when treatments commenced at symptom onset [40]. However, despite promising outcomes from the initial stages of clinical assessment, neither provided evidence for disease modification when assessed in later stage trials [41,42]. Is excitotoxicity therefore not a valid therapeutic target for treating ALS? Or is excitotoxicity in ALS perhaps a relatively late-stage event whose mitigation offers limited scope for meaningful disease-modification? Afterall, the current frontline treatment option, riluzole, purportedly targets molecular features associated with excitotoxicity [4].

Similarly, oxidative and nitrosative damage resulting from an imbalance in free radicals and endogenous protective mechanisms is a feature of all forms of ALS [43]. Free radical scavengers have therefore been assessed as a therapeutic strategy, with strong candidates including coenzyme Q_10_, creatine and edaravone all producing encouraging results when tested in mutant SOD1 mice [44,45,46,47]. Coenzyme Q_10_ and creatine both failed to produce robust outcomes when assessed in clinical trials [48,49]. Edaravone, by contrast, did produce sufficient clinical evidence to gain clinical approval in some countries [50]. Current lines of evidence, however, indicate edaravone is suitable only for a subset of patients [51], a result that seems incongruous with the seemingly broad manifestation of free radical damage in ALS.

In addition to considering pertinence of strategies that are effective in mutant SOD1 mice to cases of ALS which do not involve SOD1 mutations, rigour and reproducibility of the pre-clinical studies that supported clinical translation also require consideration. Guidelines and recommendations for rigorous pre-clinical studies [52,53,54] are becoming increasingly adopted, particularly as they are increasingly mandated by many sponsors of ALS studies, but independent reproduction of reported results is equally important. The ALS Therapy Development Institute (ALS TDI) has for many years undertaken pre-clinical studies to assess treatments which had previously been reported to generate positive outcomes. In most instances, the ALS TDI has produced outcomes to indicate that previously published positive outcomes in mutant SOD1 models are not replicable [53], suggesting that for some, subsequent negative outcomes in clinical testing could have been anticipated. This scenario, however, appears to be shifting. In 2017 the ALS TDI reported the first instance in which they were able to successfully and independently validate a previously reported positive therapeutic outcome from a pre-clinical study involving mutant SOD1 mice. The compound tested was Cu^II^(atsm) [55].

## 4. Therapeutic Efficacy of Cu^II^(atsm) in Mutant SOD1 Mouse Models of ALS

Cu^II^(atsm) (diacetylbis(4-methylthiosemicarbazonato)copper^II^) is a small molecular weight (molecular mass = 322), orally bioavailable, blood–brain barrier permeant copper-containing compound. The first reported evidence for Cu^II^(atsm) providing positive therapeutic outcomes in mutant SOD1 mice was published in 2011 [56] (Table 1). When administered to low copy number SOD1G93A mice, treatment with Cu^II^(atsm) mitigated the progressive decline in motor function, protected motor neurones in the CNS, and resulted in an overall extension in animal survival. Consistent with previously reported evidence for the compound’s ability to protect against harmful free radicals [57], CNS tissue collected from Cu^II^(atsm)-treated SOD1 mice revealed evidence for decreased protein nitration and oxidation damage [56]. Evidence for decreased astrogliosis and microgliosis were also reported [56]. Subsequent pre-clinical assessment studies spanning different experimental paradigms have corroborated these initial findings, including that of the ALS TDI [55,58,59,60,61]. Overall, the positive therapeutic outcomes generated when assessing Cu^II^(atsm) in mutant SOD1 mouse models of ALS include: improved motor function; neuroprotection; increased survival; return to disease progression upon treatment removal; dose-proportional disease modification; better therapeutic efficacy than riluzole when compared head-to-head; suitability for co-administration with riluzole; and strong disease-modification when treatments commenced after overt motor symptom onset.

Arguably the most compelling pre-clinical evidence for therapeutic efficacy in a mutant SOD1 mouse model was derived from a study in which an additional transgene was introduced to the SOD1G93A model. Median survival of standard high copy number SOD1G93A mice (on a congenic background) is around 130 days, but co-expression of the human copper chaperone for SOD (CCS) dramatically exacerbates the disease phenotype [62]. A study utilising the SOD1G93AxCCS mice demonstrated that when left untreated, median survival was ~10 days. However, when pregnant dams were treated with Cu^II^(atsm), then the pups treated commencing at 5 days old, continuous treatment with Cu^II^(atsm) resulted in most of the SOD1G93AxCCS mice surviving for over 16 months [58].

## 5. A SOD1-Specific Mechanism of Action for Cu^II^(atsm)

Some ALS-causing mutations in SOD1 affect the enzyme’s dismutase activity whereas others do not [63,64]. Copper is required for the enzyme’s dismutase activity [65]. Nascent SOD1 contains no metal ions, but through the acquisition of zinc and then copper, apo-SOD1 is converted to its physiological, metal-replete holo form. Holo-SOD1 is a highly stable protein [64]. High levels of transgene expression in SOD1 mouse models are associated with perturbations in the natural abundance of copper (and zinc), including evidence for elevated copper levels in the affected CNS [66,67]. Therapeutic strategies aimed at mitigating copper accumulation have been assessed and have produced positive outcomes [63,66,67,68,69]. However, an assessment of the metalation state of SOD1 in SOD1G37R mice revealed that SOD1 accumulates in the CNS of these animals in a copper-deficient state [59,70]. Moreover, despite ubiquitous expression of the transgene, the mutant SOD1 appears to only accumulate in a copper-deficient state in the CNS [71]. Thus, although overall SOD1 and copper levels may be elevated in the spinal cord of the transgenic animals, the disease-causing protein accumulates in an aberrant copper-deficient state. Significantly, when treated with Cu^II^(atsm) isotopically enriched with copper-65, it was demonstrated that copper administered orally to the animals as ^65^Cu^II^(atsm) entered the bioavailable pool and was ultimately incorporated into mutant SOD1 in the spinal cord [59] (Table 1). It was therefore hypothesised that treatment with Cu^II^(atsm) was protective in the mutant SOD1 mice because delivery of bioavailable copper stabilised the mutant SOD1 in a physiological holo form [59]. Corroboration of this was published in a subsequent study which specifically measured the copper state of mutant SOD1 in Cu^II^(atsm) treated mice [58].

The apparent incongruous results derived from Cu^II^(atsm) and treatments aimed at mitigating copper accumulation in the CNS may, therefore, illustrate the important role that the metalation state of SOD1 plays in its contribution to neuronal death in ALS, with both strategies preventing accumulation of an aberrant, partially metallated intermediary. Dissociation of bound copper ions from SOD1 can cause accumulation of misfolded SOD1 [69,72,73] and misfolded SOD1 is an early pathological feature in mutant SOD1 mice [74,75,76]. Cu^II^(atsm) could prevent accumulation of this aberrant intermediate by augmenting the formation of physiologically mature SOD1 through a copper delivery mechanism.

## 6. Evidence for Therapeutic Activity of Cu^II^(atsm) not Involving Mutant SOD1

As described in Section 5, a therapeutic role for Cu^II^(atsm) in mitigating neuronal decline and disease symptoms in cases of ALS caused by mutant SOD1 can potentially be attributed to the requirement for copper in SOD1. However, as described in Section 2, most cases of ALS do not involve mutant SOD1. For these cases, relevance of a therapeutic mechanism of action specifically involving the availability of copper to SOD1 is therefore unclear. For these cases, insights to the potential therapeutic utility of Cu^II^(atsm) derived from studies independent of mutant SOD1 are more pertinent. The following section of this review provides a brief overview of such studies.

### 6.1. In Vitro Evidence

The first report for therapeutic efficacy of Cu^II^(atsm) in a mutant SOD1 mouse model of ALS provided evidence for the treatment decreasing cytoplasmic levels of phosphorylated TDP-43 [56]. TDP-43 pathology is common in ALS, with mutant SOD1 cases providing a very rare exception [77]. When TDP-43 pathology is present, aberrant cytoplasmic accumulation is a conspicuous feature. Relevance of the cytoplasmic accumulation of TDP-43 reported in the SOD1 mice—and its mitigation by treatment with Cu^II^(atsm)—to ALS in humans is unclear. However, cytoplasmic accumulation of endogenous TDP-43 can be modelled in vitro by applying stressors to cells grown in culture and this is routinely performed using cells that do not express mutant SOD1. For example, treatment with mitochondrial toxins such as paraquat can induce the formation of a diverse array of features of TDP-43 pathology which are evident in ALS, including nuclear depletion, accumulation of C-terminal fragments, and accumulation in stress granules [78]. The latter of these can subsequently give rise to TDP-43 aggregation [79]. When induced in SH-SY5Y or HeLA cells, co-treatment with Cu^II^(atsm) prevented the formation of TDP-43 positive stress granules, as determined by immunofluorescence microscopy and co-localisation with the stress granule marker HuR [80] (Table 1). The mechanism of action was proposed to involve extracellular signal-regulated kinase (ERK) 1/2 signalling due to analogous results derived from cells treated with the ERK1/2 inhibitor PD98059 [78,80]. Moreover, treatment with CuCl_2_ or Cu^II^(gtsm)—which is structurally very similar to Cu^II^(atsm)—produced comparable effects on ERK1/2 and the incorporation of TDP-43 into stress granules [80], indicating that increased bioavailability of copper was involved.

Additionally, treatment with Cu^II^(atsm) promotes neurite outgrowth when applied to neurone-like PC-12 cells [81], and, more recently, has been shown to protect against the iron-dependent form of cell death known as ferroptosis [82]. Ferroptotic cell death was induced in cultured cells using a range of different pro-ferroptotic conditions and was also assessed in cell-free assays of lipid peroxidation—the toxic endpoint of ferroptosis. In all experiments Cu^II^(atsm) afforded protection comparable to the gold-standard ferroptosis inhibitor liproxstatin [82]. Iron accumulation and ferroptosis are both implicated in ALS and other neurodegenerative diseases [83] and protection against ferroptosis is therefore hypothesised as a potential treatment strategy. Although its involvement in ALS still requires further elucidation, ferroptosis is not regarded as a mutant SOD1-specific phenomenon. None of the in vitro and cell-free assays utilised when assessing the anti-ferroptotic potential of Cu^II^(atsm) involved mutant SOD1 [82]. The inhibition of ferroptosis by Cu^II^(atsm) is therefore a plausible mechanism by which Cu^II^(atsm) may provide neuroprotection in cases of ALS that do not involve mutant SOD1.

### 6.2. In Vivo Evidence

Evidence for Cu^II^(atsm) producing therapeutic outcomes in animal models of ALS involving mutations in genes other than *SOD1* have not been reported. However, a relatively recent publication does describe disease modification and neuroprotection in a toxin model of the disease [84]. Exposure to the neurotoxin β-sitosterol β-d-glucoside produced a moderate ALS-like phenotype in mice which included motor neurone loss in the spinal cord accompanied by microglial activation, neurological symptoms (loss of hind-limb extension reflex), and a decrease in motor performance. Treatment with Cu^II^(atsm) mitigated all of these ALS-like features [84], providing the first indication from an ALS-like in vivo model which does not involve mutant SOD1 for the protective activity of the treatment.

More broadly, Cu^II^(atsm) has also been assessed in mouse models pertinent to other neurological conditions. The most comprehensive of these studies relates to Parkinson’s disease. As per ALS, Parkinson’s disease is a progressive neurodegenerative disease which afflicts a confined group of neurones in the CNS, particularly dopaminergic neurones. Dopaminergic neurone loss and associated cognitive and motor deficits are modelled in mice through transgenic expression of disease related mutations (e.g., overexpressing human α-synuclein containing the A53T substitution mutation [85,86]) or toxin-induced ablation within the substantia nigra (e.g., injection of MPTP [1-methyl-4-phenyl-1,2,3,6-tetrahydropyridine] or 6-hydroxydopamine). When assessed in four different mouse models of Parkinson’s disease, treatment with Cu^II^(atsm) produced neuroprotective and therapeutic outcomes across all four models [87] (Table 1). Notably, protection against protein nitration was demonstrated, indicating a protective mechanism in the Parkinson’s disease models which was also indicated in the first study to show efficacy of Cu^II^(atsm) in a mutant SOD1 mouse model of ALS [56]. In a follow-up study, gene expression changes were assessed in brain tissue from the MPTP model using whole genome transcript analysis and the corrective effect of Cu^II^(atsm) on differentially expressed genes assessed [88]. A broad range of molecular effects supportive of the therapeutic outcomes previously reported in the MPTP mouse model of Parkinson’s disease were reported [88].

Neuroprotective and therapeutic outcomes from four different models of Parkinson’s disease confirm that the therapeutic implications of Cu^II^(atsm) are not restricted to the domain of mutant SOD1. As a further illustration of this, Cu^II^(atsm) is also protective in in vitro and in vivo models of stroke with transient and permanent ischemic injury [57,89], where Cu^II^(atsm) increases the brain copper content, reduces infarct size, and dampens inflammation markers in the ischemic brain [89]. Additionally, Cu^II^(atsm) increases copper levels in the brain and attenuates activation of microglia and astrocytes in an inflammation model involving peripheral administration of bacterial lipopolysaccharide [90]. Thus, reported outcomes supportive a therapeutic activity for Cu^II^(atsm) in animal models of neurological disease to date span 11 studies and 13 different animal models (Table 1). The majority of the reported positive outcomes involved animal models that do not involve mutant SOD1 (eight out of the 13 models).

### 6.3. Clinical Evidence

Cu^II^(atsm) labelled with radioactive isotopes of copper has been used as an imaging agent in positron-emission tomography (PET) studies. Most of these studies have focused on imaging hypoxic tissue and tumours [57,91,92] where selective retention of the tracer has been associated with mitochondrial dysfunction driving cellular retention of the copper from Cu^II^(atsm) [93,94] (Table 1). Mitochondrial dysfunction and associated oxidative stress are evident in many diverse neurological conditions [95,96] and more recent PET imaging studies are now illustrating that radiolabelled Cu^II^(atsm) is selectively retained in disease affected regions of the CNS in diverse neurological conditions. These include ALS [97], Parkinson’s disease [98] and MELAS syndrome [99] (Table 1). In the ALS study, increased retention of Cu^II^(atsm) in the ALS-affected brain was associated with a poorer score for individual patients on the ALS Functional Rating Scale (Revised), suggesting the amount of Cu^II^(atsm) retained in the affected brain was proportional to disease severity [97]. From a therapeutic perspective, however, these PET imaging studies provided a much more important outcome—they illustrated that Cu^II^(atsm) selectively targets the anatomical regions of the CNS where it would be required to exert a neuroprotective effect. Importantly, the ALS PET imaging study included sporadic cases of the disease.

More recently, a pre-print server report has presented data illustrating a broad range of changes related to the physiological requirement for copper in human, ALS-affected spinal cord tissue [100]. Quantitation of atomic copper demonstrated that the anatomical and biochemical distribution of copper is disrupted in ALS; gene expression analyses revealed that 12 out of 20 genes involved in copper handing are differentially expressed in ALS; and the assessment of diverse cuproenzymes revealed that while some cuproenzymes are increased in activity in ALS, others appear to not obtain their requisite supply of copper for optimal activity [100]. Copper dependent SOD1 activity appeared unchanged. Notably, the tissues analysed were all obtained from sporadic cases of ALS. In the context of a possible role for Cu^II^(atsm) in producing disease-modifying outcomes for patients with ALS via a mechanism involving improved copper bioavailability, these data are supportive of the pertinence of that mechanism to sporadic cases of the disease. They also indicate that there are more significant biochemical targets for this mechanism than SOD1.

## 7. Future Directions

Support for Cu^II^(atsm) as a treatment for ALS, and other neurological conditions, is already strong, to the extent that advanced clinical assessment is currently underway. However, the mechanisms by which Cu^II^(atsm) exerts its neuroprotective activity are not yet resolved. More detailed understanding of how Cu^II^(atsm) works is essential and this could be derived from lines of investigation which have not yet been explored. These could include assessment in additional animal models of ALS which are based on ALS genes other than SOD1. They could also include assessment of Cu^II^(atsm) in cell lines derived from ALS patients. Such studies could help define whether specific ALS patients may be more suited to treatment with Cu^II^(atsm) than others. They could help tailor treatment regimens on a personalised basis by, for example, identifying biomarkers which help monitor optimal response to the treatment. Additionally, more detailed investigation of Cu^II^(atsm) as a PET tracer could provide important insight to the potential for use of this compound as a theragnostic agent.

## 8. Summary

The orally bioavailable and blood–brain barrier permeant copper complex, Cu^II^(atsm), has been developed as a new treatment option for ALS. Pre-clinically, it has been extensively tested and independently validated. The more compelling outcomes for Cu^II^(atsm) as an effective treatment option are to date derived from pre-clinical studies which utilised mutant SOD1 mouse models of the disease. The therapeutic implications for Cu^II^(atsm), however, extend beyond mutant SOD1 cases of ALS (Table 1). Phase 1 assessment of Cu^II^(atsm) in ALS patients was successfully completed [101] and phase 2/3 testing is now underway [102]. Additionally, phase 1 assessment in Parkinson’s disease patients has also been completed [103]. Demonstration of effective disease modification will herald a new class of compound for the treatment of ALS and, potentially, other neurodegenerative diseases.

**Table 1:** Summary of existing clinical and pre-clinical support for Cu^II^(atsm) as a novel treatment option for amyotrophic lateral sclerosis (ALS) and other neurological conditions. * Studies highlighted with an asterisk provide outcomes which are not limited to models or cases of disease involving mutant superoxide dismutase 1 (SOD1).

Study Type	Study	Primary Outcome/Stage of Development
Pre-clinical, in vitro	* Wada et al. [57]	Protection against lipid peroxidation in isolated heart ischemia-reperfusion injury model.
	* Parker et al. [80]	Inhibition of stress-induced incorporation of TDP-43 into cytoplasmic stress granules.
	* Bica et al. [81]	Promotion of neurite outgrowth in neurone-like PC12 cells.
	* Southon et al. [82]	Protection against ferroptosis, an iron-dependent form of non-apoptotic cell death.
	* Choo et al. [90]	Reduction in markers of inflammation in stimulated primary microglia and primary astrocytes.
	* Hung et al. [87]	Protection against peroxynitrite-induced a-synuclein nitration and protection against peroxynitrite-induced cell death.
	* Huuskonen et al. [89]	Protection against oxygen-glucose deprivation in neuroblastoma N2a cells and protection against glutamate-induced excitotoxicity in primary neurones.
	* Yoshii et al. [93] and * Donnelly et al. [94]	Selective cellular retention of copper from Cu^II^(atsm) promoted by impaired electron flux through the mitochondrial electron transport chain
Pre-clinical, in vivo	Soon et al. [56]	Therapeutic efficacy in low copy number SOD1G93A mice with evidence for neuroprotection, suppression of oxidative and nitrative damage to proteins, and decreased markers of astro- and microgliosis.
	McAllum et al. [61]	Dose-proportional disease modification and greater therapeutic outcomes than riluzole in SOD1G37R mice. Evidence for disease modification when treatment started at a late stage of motor function deficit.
	Roberts et al. [59]	Demonstration of in vivo transfer of copper from orally administered Cu^II^(atsm) to copper deficient SOD1 in spinal cord of SOD1G37R mice.
	Williams et al. [58]	Demonstration of long-term therapeutic efficacy in the rapidly fatal CCSxSOD1G93A mouse model and corroboration of Cu^II^(atsm) improving the copper metalation state of SOD1 in vivo.
	Hilton et al. [60]	Therapeutic efficacy in SOD1G93A mice on mixed genetic background. Evidence for suppression of oxidative damage to proteins, decreased markers of astro- and microgliosis, and improved SOD1 activity.
	Vieira et al. [55]	First independent validation of an ALS drug candidate reported by the ALS Therapy Development Institute. Corroborated therapeutic efficacy of Cu^II^(atsm) in SOD1G93A mice on mixed genetic background.
	* Kuo et al. [84]	Cu^II^(atsm) is protective in toxin model of ALS. Treatment preserved motor neurones in the spinal cord, improved motor function and decreased microglial activation.
	* Hung et al. [86]	Neuroprotection and improved cognitive and locomotive function in four mouse models of Parkinson’s disease (α-synA53T, MPTP, 6-OHDA, and α-synA53T + MPTP).
	* Huuskonen et al. [89]	Neuroprotection and decreased lesion volume in transient and permanent models of cerebral ischemia.
	* Choo et al. [90]	Reduction in acute cerebrovascular inflammation caused by peripheral administration of bacterial lipopolysaccharide.
	* Cheng et al. [88]	Restoration of expression of diverse genes in MPTP mouse model of Parkinson’s disease determined via whole transcriptome sequencing.
Clinical	* Ikawa et al. [97]	PET imaging showed selective accumulation of Cu^II^(atsm) in disease-affected region of brain in ALS patients.
	* Ikawa et al. [98]	PET imaging showed selective accumulation of Cu^II^(atsm) in disease-affected region of brain in PD patients.
	* Ikawa et al. [99]	PET imaging showed selective accumulation of Cu^II^(atsm) in disease-affected region of brain in MELAS patients.
	* NCT02870634 [101]	Phase 1 dose escalation and study of Cu^II^(atsm) in ALS/MND patients.
	* NCT04082832 [102]	Phase 2/3 study of Cu^II^(atsm) compared with placebo for treatment of ALS/MND.
	* NCT03204929 [103]	Phase 1 dose escalation study of Cu^II^(atsm) in Parkinson’s disease patients.
